# Performance of the National Early Warning Score 2 for diagnosing bacterial sepsis: a prospective single-center study in Vietnam

**DOI:** 10.62838/jccm-2026-0047

**Published:** 2026-10-30

**Authors:** Le Buu Chau, Nguyen Dinh Thuy Thuy, Nguyen Phuong Thao, Nguyen Thi Kim Ngan, Dang Van Tri

**Affiliations:** Department of Infectious Diseases, School of Medicine, University of Medicine and Pharmacy at Ho Chi Minh City, Ho Chi Minh City, Vietnam; Hospital for Tropical Diseases, Ho Chi Minh City, Vietnam; Department of Infectious Diseases, Military Hospital 175, Ho Chi Minh City, Vietnam; Emergency Department, University Medicine Center Ho Chi Minh City, Ho Chi Minh City, Vietnam

**Keywords:** sepsis, infectious diseases, early warning score, sensitivity and specificity, receiver operating characteristics

## Abstract

**Background:**

Early recognition of sepsis is vital for enhancing patient outcomes. While the National Early Warning Score 2 (NEWS2) has demonstrated good performance for identifying sepsis in emergency departments, its diagnostic accuracy in other clinical environments remains uncertain. This study aimed to evaluate the diagnostic accuracy of NEWS2 for bacterial sepsis at an infectious diseases department.

**Methods:**

This prospective diagnostic accuracy study consecutively enrolled adult patients admitted to the Department of Infectious Diseases (DID), Military Hospital 175, Vietnam, between March and July 2025. Eligible patients were aged ≥18 years and had suspected bacterial infection. NEWS2 was assessed within the first 24 hours of hospitalization. For patients admitted from the Emergency Department, NEWS2 was calculated using the initial clinical data recorded there; for those admitted from the Outpatient Clinic, it was assessed on admission to the DID. Sepsis was defined according to Sepsis-3 criteria. Diagnostic performance was evaluated using the area under the receiver operating characteristic curve (AUROC), sensitivity, and specificity.

**Results:**

Of 741 patients screened, 120 were included in the final analysis, 83 patients (69.2%) were diagnosed with sepsis, including 1 case of septic shock. NEWS2 demonstrated moderate discrimination for identifying sepsis, with an AUROC of 0.75 (95% confidence interval: 0.66–0.84). The optimal cut-off was ≥5, yielding a sensitivity of 71.1% and a specificity of 73.0%.

**Conclusions:**

NEWS2 demonstrated moderate diagnostic performance for identifying sepsis in this setting and may serve as a useful adjunctive tool for early risk stratification, particularly in resource-limited infectious diseases departments.

## Introduction

Sepsis is characterized by life-threatening organ dysfunction resulting from a dysregulated host response to infection [1]. This illness remains a major global health concern despite significant progress in health-care, especially in low- and middle-income countries [2]. Globally, Rudd et al. estimated that the annual incidence of sepsis and sepsis-related mortality reached approximately 48.9 million and 11.0 million cases, respectively [3]. Consequently, early identification and prompt initiation of appropriate antimicrobial therapy are considered crucial for improving patient prognosis and clinical outcomes [4,5].

Since 2016, the Sequential Organ Failure Assessment (SOFA) has been integrated into the diagnostic criteria for sepsis [1]. Nevertheless, the use of the SOFA score necessitates several laboratory tests, which can delay clinical assessment while awaiting results. To address these limitations, the quick Sequential Organ Failure Assessment (qSOFA) was introduced as a rapid, supplementary screening tool to facilitate early detection of patients with suspected infection who are at risk of clinical deterioration [1]. Nevertheless, the Surviving Sepsis Campaign Guidelines 2021 advise against utilizing the qSOFA as a screening instrument for sepsis due to its limited sensitivity and the potential to miss critically ill patients [6]. This constraint underscores the necessity to explore alternative screening methods with enhanced diagnostic efficacy.

In England, the National Early Warning Score 2 (NEWS2) [7] and its previous version, the National Early Warning Score (NEWS) [8], serve as a standardized universal language within the National Health Service for delineating illness severity in acutely ill patients and enhancing the detection of clinical deterioration [9]. The clinical utility of the NEWS and NEWS2 has predominantly been evaluated in emergency care settings [10]. Oi et al. reported an area under the receiver operating characteristic curve (AUROC) of 0.846 (95% confidence interval [CI]: 0.756–0.937) for NEWS in predicting sepsis [11]. Compared with the qSOFA, NEWS2 demonstrated superior performance in sepsis prediction [12]. In contrast, a study conducted by Oduncu et al. found no statistically significant difference in AUROC between NEWS and qSOFA for sepsis diagnosis (p = 0.43) [13].

In addition, research evaluating the discrimination of NEWS and NEWS2 beyond emergency settings—particularly within infectious disease departments, where patients with suspected infections are often managed—remains limited. Therefore, this study aimed to assess the diagnostic performance of NEWS2 for sepsis among patients with suspected bacterial infection admitted to an infectious diseases department in southern Vietnam. This study was reported in accordance with the Standards for Reporting Diagnostic Accuracy Studies guidelines.

## Materials and Methods

### 2.1. Study setting

This study was conducted at the Department of Infectious Diseases (DID), Military Hospital 175, Vietnam, from March 1 to July 31, 2025. The department is equipped with 70 beds and provides care for patients with a wide spectrum of infectious diseases, including dengue fever, viral hepatitis, influenza, bloodstream infections, respiratory tract infections, urinary tract infections, gastrointestinal infections, skin and soft tissue infections, and meningitis.

Patients were admitted to the DID through two pathways. First, patients could be admitted directly from the Outpatient Clinic (OPC). Second, patients with unstable clinical conditions were initially admitted to the Emergency Department (ED), where triage and severity assessment were performed; they were then transferred to the DID or other appropriate departments.

### 2.2. Study design and participants

This was a prospective diagnostic accuracy study with consecutive recruitment. Adult patients admitted to the DID were screened for eligibility. Inclusion criteria were age ≥18 years and suspected bacterial infection [14]. Patients were excluded if they were pregnant, had a history of spinal cord injury, had received treatment at another hospital within the previous 48 hours, or had a hospital stay of ≤ 24 hours. Eligible patients were enrolled after informed consent had been obtained from the patient or a legal representative.

The NEWS2 and sepsis assessments were conducted within the first 24 hours of hospitalization. For patients admitted from the ED, the NEWS2 was calculated using the initial clinical data documented in the ED to minimize potential bias. For patients admitted from the OPC, the NEWS2 was assessed upon immediate admission to the DID. Although no formal blinding procedure was implemented between NEWS2 and sepsis assessments, the assessors were not involved in treatment decisions.

### 2.3. Study variables

The NEWS2 is derived from seven physiological parameters: (1) respiratory rate, (2) peripheral oxygen saturation (SpO_2_), (3) requirement for supplemental oxygen, (4) body temperature, (5) systolic blood pressure, (6) heart rate, and (7) level of consciousness. The total score varies from 0 to 20 points [7].

The SOFA is calculated based on six organ systems: (1) respiratory, (2) coagulation, (3) hepatic, (4) cardiovascular, (5) renal, and (6) neurological function. The total score varies from 0 to 24 points [1].

The qSOFA is assessed using three clinical parameters: (1) altered mental status, (2) systolic blood pressure ≤100 mmHg, and (3) respiratory rate ≥22 breaths per minute. A qSOFA is considered positive when at least two of the three criteria are met [1].

Patients were considered to have a suspected bacterial infection if intravenous antimicrobial therapy was initiated concurrently with any sampling for culture [14].

Sepsis was defined as a suspected infection [14] accompanied by an acute increase in the SOFA of at least 2 points. Septic shock was regarded as a subset of sepsis and was diagnosed when patients necessitated vasopressor therapy to sustain a mean arterial pressure ≥65 mmHg and had a serum lactate level >2 mmol/L [1].

### 2.4. Sample size

The sample size was estimated based on the AUROC using the method described by Hanley and McNeil. Sample size calculation was performed using Med-Calc (MedCalc Software Ltd, Belgium). Considering a case-to-control ratio of 1.6 and an expected AUROC of 0.73 based on the study of Oduncu et al. [13], the minimum required sample sizes were 39 cases in the disease group and 24 in the control group (type I error of 0.05 and type II error of 0.1).

### 2.5. Statistical analysis

Categorical variables were reported as numbers with percentages. Continuous variables were presented as median (interquartile range [IQR]) or mean (standard deviation [SD]) for variables with normal distribution. Comparisons between groups were performed using the χ^2^ test, Fisher’s exact test, Student’s t-test, or Mann–Whitney U test, as appropriate. A p-value <0.05 was considered statistically significant.

The AUROC was used to evaluate the diagnostic performance of NEWS2 for sepsis. We identified the optimal cut-off value based on the Youden J index. Comparisons between two AUROCs were conducted using the DeLong test. In addition, sensitivity, specificity, positive likelihood ratio, and negative likelihood ratio were also calculated. A sensitivity analysis was conducted to assess the robustness of the main findings in the subset of patients with complete PaO_2_ data.

Model calibration was assessed using the Hosmer-Lemeshow test, with a p-value >0.05 indicating adequate fit. Internal validation of the multivariable model was performed using bootstrap resampling with 1000 repetitions to assess the stability of the regression estimates. A multivariable logistic regression model was constructed to assess the independent association between NEWS2 and sepsis, adjusting for age, sex, ED admission, and the presence of any comorbidity.

Stata (version 17.0, Stata Corp LLC, USA) was used to perform the statistical analyses. The receiver operating characteristic (ROC) curve was plotted in RStudio (version 2025.05.0; Posit Software, USA) using the pROC package.

### 2.6. Ethics statement

The study was approved by the Institutional Review Board of Military Hospital 175, Ho Chi Minh City, Vietnam (Approval No. 792/GCN-HDDD). Written informed consent was obtained from all participants prior to enrollment in the study.

## RESULTS

Between March and July 2025, 741 patients were admitted to the DID and screened for eligibility. Among them, 315 patients met the inclusion criteria. Of the 315 eligible patients, 195 were excluded, leaving 120 for the final analysis (Figure 1).

**Figure 1.**
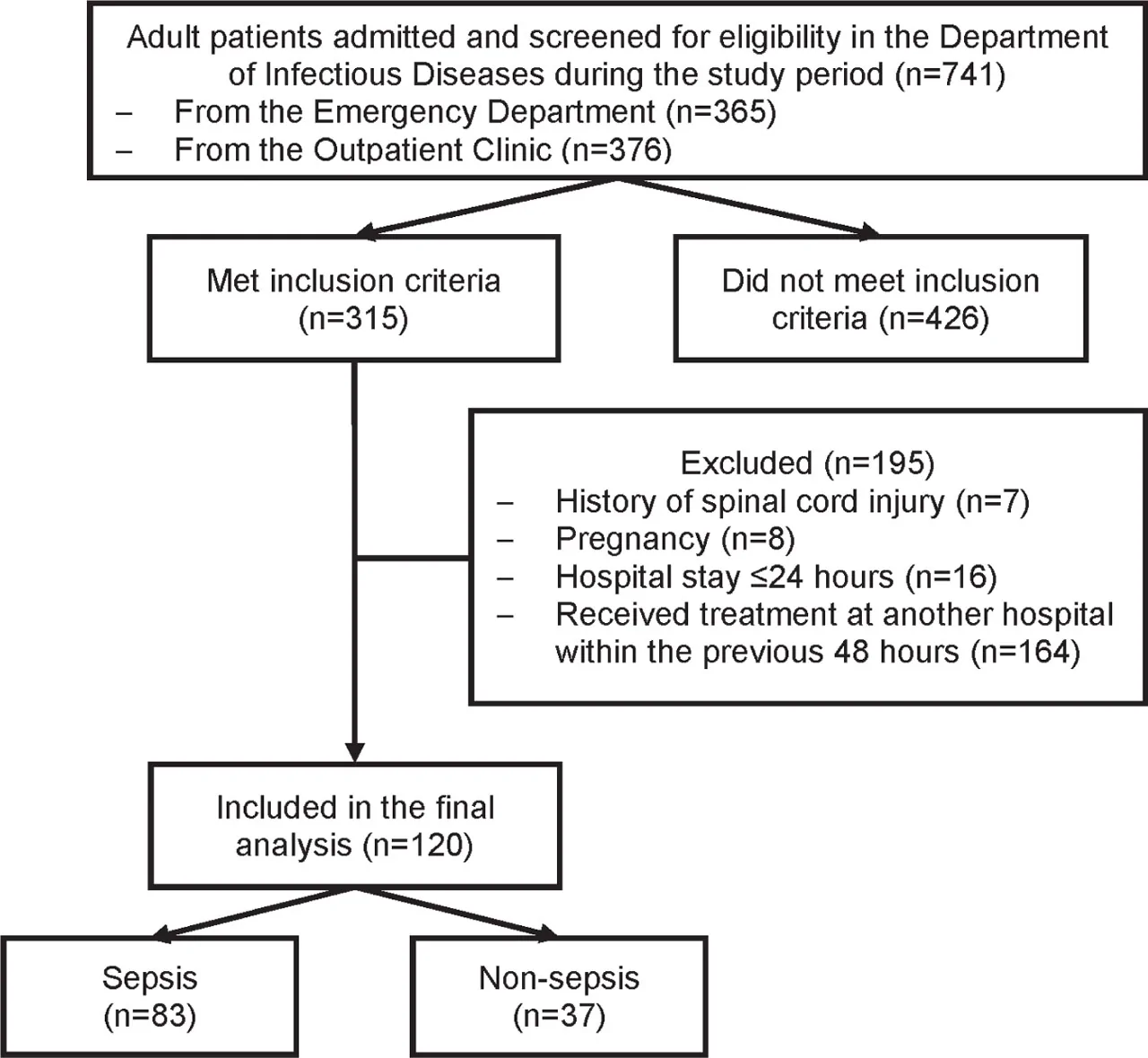
Flow diagram of patient recruitment

Among patients with sepsis, 52 (62.7%) were admitted from the ED and 31 (37.3%) from the OPC. In the non-sepsis group, 12 (32.4%) and 25 (67.6%) patients were admitted from the ED and OPC, respectively.

### 3.1. Characteristics of the study population

Table 1 presents a comparison of baseline characteristics according to sepsis diagnosis. Patients with sepsis had a significantly higher median age, were more frequently admitted via the ED, and were more likely to have at least one comorbidity than patients without sepsis (p <0.001, p = 0.002, and p = 0.003, respectively). No difference in sex distribution was observed between the two groups (p = 0.337). Regarding laboratory parameters, patients with sepsis had significantly lower platelet counts and higher serum creatinine, total bilirubin, and procalcitonin levels compared to those without sepsis (all p <0.05).

**Table 1.** Baseline characteristics of patients with and without sepsis

**Characteristic**	**Sepsis (n = 83)**	**Non-sepsis (n = 37)**	p-value^*^
**Demographic characteristics**			
Age, years, median (IQR)	71 (60–77)	62 (40–68)	<0.001
Male sex, n (%)	37 (44.6)	20 (54.1)	0.337
Admission from ED, n (%)	52 (62.7)	12 (32.4)	0.002
**Comorbidities**			
Presence of any comorbidity, n (%)	77 (92.8)	27 (73.0)	0.003
Cardiovascular disease^§^, n (%)	60 (72.3)	19 (51.4)	0.026
Diabetes mellitus, n (%)	37 (44.6)	12 (32.4)	0.211
Corticosteroid use, n (%)	18 (21.7)	5 (13.5)	0.294
Other comorbid conditions^†^, n (%)	15 (18.1)	3 (8.1)	0.158
**Foci of infection**			
Gastrointestinal tract infection, n (%)	24 (28.9)	9 (24.3)	0.603
Urinary tract infection, n (%)	26 (31.3)	9 (24.3)	0.436
Respiratory tract infection, n (%)	20 (24.1)	6 (16.2)	0.333
Other foci of infection^¶^, *n(%)*	13 (15.7)	13 (35.1)	0.017
**Laboratory findings**			
White blood cell count, ×10^9^/L, median (IQR)	11.5 (8.5–16.8)	14.4 (8.8–18.4)	0.269
Hemoglobin, g/L, median (IQR)	121 (105–139)	132 (121–139)	0.072
Platelet count, ×10^9^/L, median (IQR)	178 (120–295)	260 (185–311)	0.008
Serum creatinine, µmol/L, median (IQR)	119 (84–158)	83 (72–92)	<0.001
Total bilirubin, µmol/L, median (IQR)	17 (11–27)	15 (11–18)	0.036
C-reactive protein, mg/L^††^, mean ± SD	107.6 ± 79.2	120.9 ± 75.5	0.649
Procalcitonin, ng/mL^††^, median (IQR)	7.4 (1.1–28.3)	1.2 (0.6–3.1)	0.004
Arterial lactate, mmol/L^††^, median (IQR)	2.6 (2.1–4.1)	2.4 (1.7–3.0)	0.175
Positive blood culture^€^, n (%)	16 (19.3)	7 (18.9)	0.963
**Clinical scores**			
NEWS2, median (IQR)	5 (4–7)	3 (2–5)	<0.001
qSOFA ≥2, n (%)	17 (20.5)	3 (8.1)	0.093

Abbreviations: ED, Emergency Department; IQR, interquartile range; SD, standard deviation; NEWS2, National Early Warning Score 2; qSOFA, quick Sequential Organ Failure Assessment.

* Continuous variables were compared using the Mann–Whitney U test or Student’s t-test; categorical variables using the χ^2^ test or Fisher’s exact test as appropriate.

§ Cardiovascular disease included hypertension, heart failure, chronic coronary disease, and/or prosthetic heart valve.

† Other comorbid conditions included chronic kidney disease (n = 12), chronic liver disease (n = 5), and human immunodeficiency virus infection (n = 1).

¶ Other foci of infection included skin and soft tissue infections (n = 14), bacterial meningitis (n = 3), and unknown foci (n = 9).

†† C-reactive protein was available in 29 patients; procalcitonin was available in 78 patients; arterial lactate was available in 75 patients who underwent arterial blood gas analysis, because these tests were not routinely performed in clinical practice at our hospital.

€ Positive blood cultures yielded *Escherichia coli* (n = 8), *Staphylococcus aureus* (n = 4), *Burkholderia pseudomallei* (n = 4), *Klebsiella pneumoniae* (n = 3), *Salmonella specie*s (n = 2), *Streptococcus pyogenes* (n = 1); and concurrent growth of* E. coli* and *K. pneumoniae* (n = 1).

In the sepsis group, 78 patients (94.0%) recovered; one patient (1.2%) was transferred to the intensive care unit for further management; and four patients (4.8%) were discharged against medical advice after exceeding 24 hours of hospitalization. In contrast, all patients in the non-sepsis group recovered.

### 3.2. Diagnostic performance of the NEWS2 for sepsis diagnosis

In our study, the NEWS2 demonstrated an AUROC of 0.75 (95% CI: 0.66–0.84) for detecting sepsis (Figure 2).

Table 2 exhibits the diagnostic performance of the NEWS2 across various cut-off values. As the cut-off point increased, specificity enhanced while sensitivity diminished. A NEWS2 cut-off of ≥5 was identified as the optimal threshold for diagnosing sepsis, with a sensitivity of 71.1% and a specificity of 73.0% (Youden J = 0.44).

**Figure 2.**
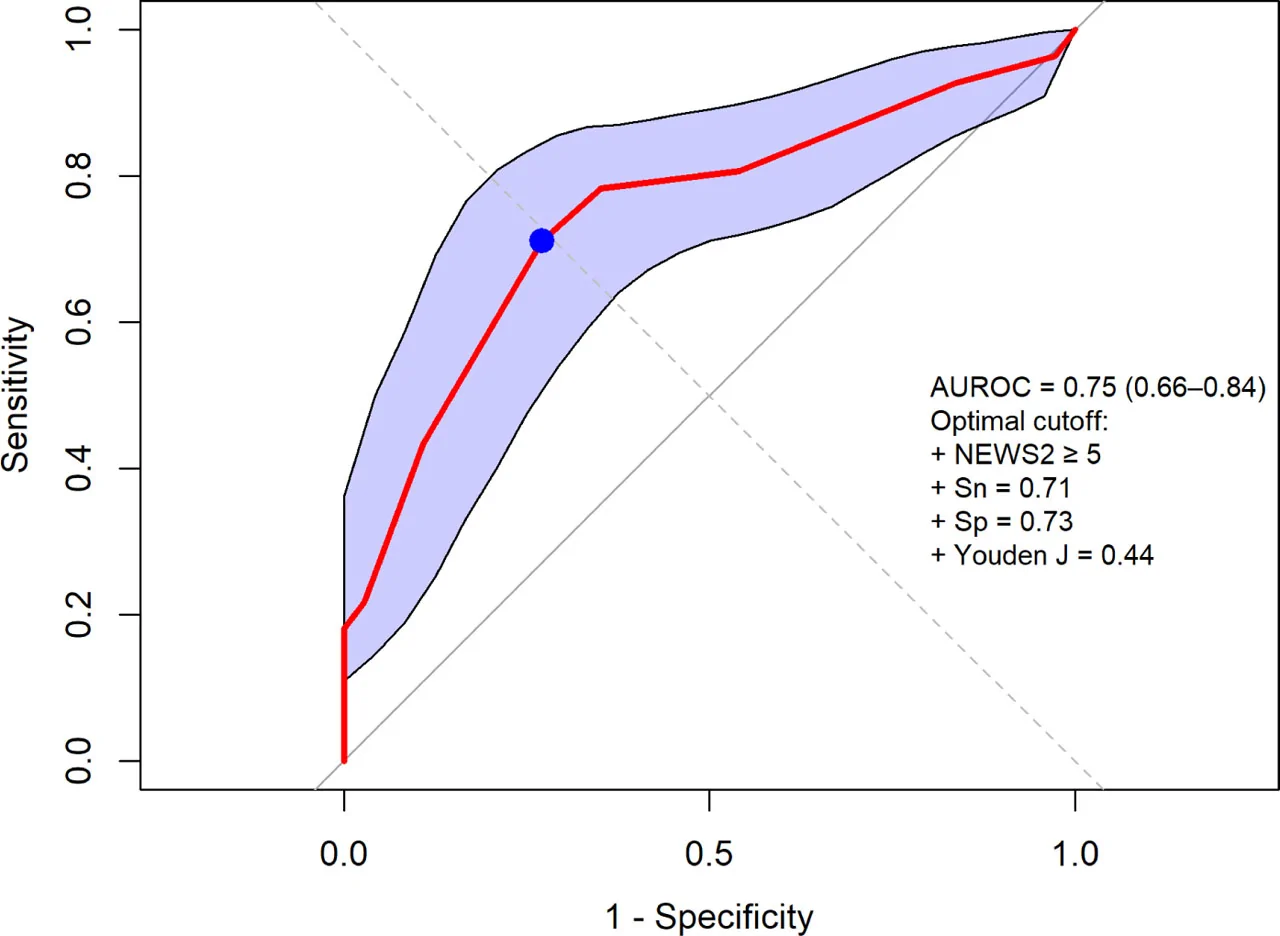
Receiver operating characteristic curve of the NEWS2 for sepsis detection. Abbreviations: AUROC, area under the receiver operating characteristic curve; NEWS2, National Early Warning Score 2; Sn, sensitivity; Sp, specificity.

**Table 2.** Diagnostic performance of the NEWS2 for discriminating sepsis at different cut-off values

**Cut-off**	**Sn (95% CI)**	**Sp (95% CI)**	**LR+ (95% CI)**	**LR− (95% CI)**
≥1	96.4 (89.8–99.2)	2.7 (0.1–14.2)	1.0 (0.9–1.1)	1.3 (0.1–12.4)
≥2	92.8 (84.9–97.3)	16.2 (6.2–32.0)	1.1 (1.0–1.3)	0.5 (0.2–1.3)
≥3	80.7 (70.6–88.6)	46.0 (29.5–63.1)	1.5 (1.1–2.1)	0.4 (0.2–0.7)
≥4	78.3 (67.9–86.6)	64.9 (47.5–79.8)	2.2 (1.4–3.5)	0.3 (0.2–0.5)
≥5	71.1 (60.1–80.5)	73.0 (55.9–86.2)	2.6 (1.5–4.5)	0.4 (0.3–0.6)
≥6	43.4 (32.5–54.7)	89.2 (74.6–97.0)	4.0 (1.5–10.5)	0.6 (0.5–0.8)
≥7	28.9 (19.5–39.9)	94.6 (81.8–99.3)	5.4 (1.33–21.47)	0.8 (0.6–0.9)
≥8	21.7 (13.4–32.1)	97.3 (85.8–100)	8.0 (1.11–57.9)	0.8 (0.7–0.9)

**Abbreviations:** Sn, sensitivity; Sp, specificity; LR+, positive likelihood ratio; LR−, negative likelihood ratio; CI, confidence interval.

### 3.3. Comparative analysis of the performance of NEWS2 and comparator models for sepsis detection

Table 3 shows the discriminative performance of NEWS2 and the comparator models for sepsis detection. The NEWS2 model demonstrated acceptable discrimination, with an AUROC of 0.75 (95% CI: 0.66–0.84). In the sensitivity analysis restricted to the 75 patients with available PaO_2_ data, NEWS2 yielded a comparable AUROC of 0.70 (95% CI: 0.58–0.82). The qSOFA model had a similar performance (AUROC = 0.73, 95% CI: 0.64–0.82). No statistically significant differences in AUROC were found between NEWS2 and qSOFA (DeLong test, p = 0.662).

**Table 3.** Discriminative performance and calibration of NEWS2 and comparator models for sepsis detection

**Model**	**n**	**H-L test ^§^**	**AUROC**	**95% CI**	**p-value^†^**
**NEWS2^¶^**					
All patients	120	0.36	0.75	0.66–0.84	-
Patients with PaO_2_ availability	75	0.75	0.70	0.58–0.82	
Patients admitted from the ED	64	0.80	0.79	0.67–0.90	
Patients admitted from the OPC	56	0.08	0.70	0.56–0.84	
**Other models**					
qSOFA^¶^	120	0.07	0.73	0.64–0.82	0.662
NEWS2 + age + sex + admission from ED + presence of any comorbidity	120	0.51	0.82	0.74–0.90	0.025

**Abbreviations:** AUROC, area under the receiver operating characteristic curve; CI, confidence interval; ED, Emergency Department; H-L, Hosmer-Lemeshow; NEWS2, National Early Warning Score 2; OPC, Outpatient Clinic; qSOFA, quick Sequential Organ Failure Assessment.

§ Hosmer-Lemeshow test results are expressed as p-values; p >0.05 indicates an adequate model fit.

† P-values were calculated using the DeLong test, with “−” as the reference; p <0.05 indicates a statistically significant difference in AUROC.

¶ NEWS2 and qSOFA were analyzed as continuous variables.

In the multivariable logistic regression model combined with age, sex, ED admission, and the presence of any comorbidity (combined NEWS2 model), higher NEWS2 was independently associated with sepsis (adjusted odds ratio [aOR] = 1.54, 95% CI: 1.25–1.90, p <0.001). Increasing age was also significantly associated with sepsis (aOR = 1.05, 95% CI: 1.01–1.08, p = 0.007). In contrast, sex (aOR = 0.79, 95% CI: 0.30–2.05, p = 0.625), admission from ED (aOR = 1.22, 95% CI: 0.48–3.13, p = 0.673), and the presence of any comorbidity (aOR = 2.11, 95% CI: 0.42–10.64, p = 0.367) were not significantly associated with sepsis. Notably, the adjusted NEWS2 model demonstrated a good performance with an AUROC of 0.82 (95% CI: 0.74–0.90), which was significantly higher than that of the NEWS2-only model (DeLong test, p = 0.025).

Bootstrap validation with 1000 replications demonstrated stable regression estimates. Higher NEWS2 (aOR = 1.54, 95% CI: 1.22–1.93, p <0.001) and increasing age (aOR = 1.05, 95% CI: 1.01–1.09, p = 0.010) remained independently associated with sepsis, whereas sex, admission from ED, and the presence of any comorbidity were not significantly associated with the outcome. The bootstrap-derived confidence intervals were consistent with the original estimates.

## DISCUSSION

In Vietnam, a lower middle-income country [15], communicable diseases constituted 13.1% of the overall disease burden in 2023 [16]. Consequently, the majority of hospitals across the nation maintain specialized settings dedicated to infectious diseases. Patients with suspected infections who are at risk of developing sepsis are generally managed in these departments. A multi-center study conducted across 15 hospitals in Vietnam reported an in-hospital mortality rate of 40.1% among patients with sepsis [17]. These findings underscore the need to identify effective tools for early recognition of sepsis in patients with suspected infection.

In addition to early warning scores such as NEWS2, several biomarkers—including procalcitonin, presepsin, interferon λ3, and bioactive adrenomedullin—have been evaluated for the early identification of sepsis [18]. Contenti et al. reported that procalcitonin and presepsin demonstrated moderate diagnostic performance for predicting sepsis and septic shock, with AUROCs of 0.711 (95% CI: 0.660–0.758) and 0.709 (95% CI: 0.658–0.756), respectively [19]. Other molecular biomarkers have also been explored; for instance, the expression level of CD86 has been identified as an independent predictor of sepsis (aOR = 1.22, 95% CI: 1.04–1.44, p = 0.001) [20]. Nonetheless, measuring these biomarkers often requires high-tech facilities, which may not be readily accessible in resource-limited settings. Consequently, simple clinical tools such as NEWS2 remain highly relevant for the early identification of sepsis in routine clinical practice.

The NEWS2 yielded an AUROC of 0.75 (95% CI: 0.66–0.84) for identifying sepsis, indicating moderate discriminatory ability. Our findings are consistent with the previous studies [13,21]. However, this performance appears slightly inferior to that of certain earlier studies, in which AUROC generally ranged from 0.80 to 0.85 [11,12]. In our study, we did not observe a statistically significant difference between NEWS2 and qSOFA in detecting sepsis (DeLong test, p = 0.662). Conversely, Mellhammar et al. reported superior performance of NEWS2 compared with qSOFA in identifying sepsis [12].

We identified a NEWS2 cut-off of ≥5 as the optimal threshold for diagnosing sepsis, with balanced sensitivity (71.1%) and specificity (73.0%). This finding is consistent with the recommendation of the Royal College of Physicians, which advises that a NEWS2 of ≥5 should prompt consideration of sepsis and trigger an urgent clinical response [7]. Nonetheless, Lam et al. reported that a NEWS2 threshold of ≥3 was the optimal cut-off, with sensitivity and specificity of 77.0% and 61.0%, respectively. At a cut-off of NEWS2 ≥5, the sensitivity decreased to 45%, suggesting that this threshold may be less suitable for sepsis screening in that study [21].

In the sensitivity analysis involving 75 patients with available PaO2 data, the AUROC was 0.70 (95% CI: 0.58–0.82), marginally lower than that observed in the full dataset with an AUROC of 0.75 (95% CI: 0.66–0.84). Nevertheless, the overlapping confidence intervals indicated that the overall results remained largely consistent.

Several factors may account for the differences between our findings and those of previous studies. The study setting and patient spectrum likely played an important role. Our study was conducted in an infectious diseases department and included 69.2% of patients diagnosed with sepsis. This proportion appears to be higher than that reported in previous studies conducted in emergency department settings, in which the proportion ranged from 27.6% to 64.0% [11,21].

Furthermore, in emergency department settings, patients are frequently evaluated at an earlier and more acute phase of illness, exhibiting greater physiological instability. As a result, abnormalities detected by NEWS2 may be more prominent, thereby enhancing its discriminatory effectiveness for sepsis. In contrast, our study encompassed patients admitted from both the ED and the OPC, leading to a more heterogeneous study population compared to previous research.

Although AUROC is generally considered less dependent on disease prevalence than predictive values, variation in sepsis prevalence may reflect underlying differences in case mix, disease severity, and patient selection. In our cohort, the high prevalence of sepsis may indicate a population with a greater pretest probability of infection-related organ dysfunction, which could have narrowed the physiological differences between patients with and without sepsis and partly explain the more moderate AUROC observed in our study.

The observed performance of NEWS2 may also be interpreted in light of the inherent limitations of the score itself. The NEWS2 is based solely on seven physiological parameters and does not incorporate demographic or baseline patient information. Although these parameters can be rapidly gathered upon hospital admission, factors such as age, sex, race, income, education level, and comorbidities may impact the epidemiology of sepsis [22].

We observed that adding clinical variables, including age, sex, ED admission, and the presence of any comorbidity, to the NEWS2 model improved its diagnostic performance with an AUROC of 0.82 (95% CI: 0.74–0.90). This improvement was statistically significant compared with the NEWS2-only model (DeLong test, p = 0.025). Notably, bootstrap resampling with 1000 replications demonstrated stable estimates for the main predictors, particularly NEWS2 and age, despite the relatively limited sample size. The minimal bias and consistent confidence intervals suggest that the model was not substantially influenced by random sampling variation, thereby supporting the robustness of the observed associations.

In our study, increasing age was identified as an independent risk factor for sepsis (aOR = 1.05; 95% CI: 1.01–1.08, p = 0.007). Indeed, sepsis disproportionately affects older adults, with more than 60% of cases occurring in individuals aged ≥65 years [23]. Several age-related physiological changes may contribute to this association, including immunosenescence, a higher burden of comorbidities, and reduced organ reserve [24]. Immunosenescence refers to the progressive decline in both innate and adaptive immune responses, which may impair the host’s ability to recognize and eliminate pathogens [25].

Moreover, Ginde et al. reported that inflammatory biomarkers tend to be elevated in older patients with sepsis compared with younger individuals [26]. Collectively, these findings suggest that age may play an important role in the pathophysiology and epidemiology of sepsis and should therefore be considered when developing or applying sepsis predictive models.

In contrast to previous findings [23,27], sex was not associated with sepsis in our study. Sex hormones have been suggested to influence immune responses. In females, estrogen may modulate the innate immune system by enhancing the activity of macrophages and lymphocytes while limiting excessive release of pro-inflammatory cytokines [27]. Although menopausal status was not directly assessed in our study, the relatively high mean age of female participants suggests that many were likely postmenopausal, which may partly explain why a protective effect of female sex was not clearly observed in our study population.

Overall, our findings suggest that NEWS2 has moderate diagnostic accuracy for sepsis among patients admitted to the infectious diseases department. Although its discriminatory performance was not superior to qSOFA in our cohort, NEWS2 still demonstrated clinically acceptable performance and identified an optimal threshold consistent with current recommendations. These results support the potential usefulness of NEWS2 as an early risk stratification tool in this setting. Nonetheless, its performance may vary across clinical contexts and patient populations, and it should therefore be interpreted alongside clinical judgment rather than used in isolation.

This study has several limitations. First, it was conducted at a single center, which may limit the generalizability of the findings. Second, the study population was heterogeneous, and patients admitted from the ED may have undergone prior triage, potentially contributing to spectrum bias. Third, no formal blinding was implemented between NEWS2 and sepsis assessments during patient enrollment, which may have introduced selection bias. Finally, the analysis focused solely on NEWS2 and did not incorporate additional biomarkers that might enhance sepsis detection.

## Conclusions

This study provides further evidence that NEWS2 may serve as a valuable adjunctive tool for the early recognition and risk stratification of sepsis in patients with suspected bacterial infection admitted to infectious diseases departments. Its reliance on routinely collected physiological parameters supports its potential applicability in resource-limited settings. Future refinement through the integration of additional patient characteristics may further enhance its clinical utility. External validation in larger multicenter cohorts is needed to confirm these findings and determine the optimal role of NEWS2 in sepsis detection.

## References

[1] Singer M, Deutschman CS, Seymour CW (2016). The Third International Consensus Definitions for Sepsis and Septic Shock (Sepsis-3). Jama.

[2] La Via L, Sangiorgio G, Stefani S (2024). The Global Burden of Sepsis and Septic Shock. Epidemiologia (Basel).

[3] Rudd KE, Johnson SC, Agesa KM (2020). Global, regional, and national sepsis incidence and mortality, 1990–2017: analysis for the Global Burden of Disease Study. Lancet.

[4] Gauer R, Forbes D, Boyer N (2020). Sepsis: Diagnosis and Management. Am Fam Physician.

[5] Timbrook TT, Spivak ES, Hanson KE (2018). Current and Future Opportunities for Rapid Diagnostics in Antimicrobial Stewardship. Med Clin North Am.

[6] Oczkowski S, Alshamsi F, Belley-Cote E (2022). Surviving Sepsis Campaign Guidelines 2021: highlights for the practicing clinician. Pol Arch Intern Med.

[7] Royal College of Physicians (GB) (2017). National Early Warning Score (NEWS) 2: Standardising the assessment of acute-illness severity in the NHS. Updated report of a working party.

[8] Royal College of Physicians (GB) (2012). National Early Warning Score (NEWS): Standardising the assessment of acute-illness severity in the NHS. Report of a working party.

[9] Williams B (2022). The National Early Warning Score: from concept to NHS implementation. Clin Med (Lond).

[10] Sabir L, Ramlakhan S, Goodacre S (2022). Comparison of qSOFA and Hospital Early Warning Scores for prognosis in suspected sepsis in emergency department patients: a systematic review. Emerg Med J.

[11] Oi Y, Ogawa F, Honzawa H, Abe T, Imaki S, Takeuchi I (2025). A Screening Tool to Predict Sepsis in Patients With Suspected Infection in the Emergency Department. Cureus.

[12] Mellhammar L, Linder A, Tverring J (2019). NEWS2 is Superior to qSOFA in Detecting Sepsis with Organ Dysfunction in the Emergency Department. J Clin Med.

[13] Oduncu AF, Kıyan GS, Yalçınlı S (2021). Comparison of qSOFA, SIRS, and NEWS scoring systems for diagnosis, mortality, and morbidity of sepsis in emergency department. Am J Emerg Med.

[14] Seymour CW, Liu VX, Iwashyna TJ (2016). Assessment of Clinical Criteria for Sepsis: For the Third International Consensus Definitions for Sepsis and Septic Shock (Sepsis-3). Jama.

[15] World Bank (2024). Vietnam [Internet].

[16] Our World in Data (2023). Share of disease burden from communicable diseases vs. GDP [Internet].

[17] Do SN, Dao CX, Nguyen TA (2023). Sequential Organ Failure Assessment (SOFA) Score for predicting mortality in patients with sepsis in Vietnamese intensive care units: a multicentre, cross-sectional study. BMJ Open.

[18] Lee GH, Kim H, Moon HW, Yun YM, Lee S, Hur M (2026). Multi-Marker Approach in Sepsis: A Clinical Role Beyond SOFA Score. Medicina (Kaunas).

[19] Contenti J, Occelli C, Lemoel F, Ferrari P, Levraut J (2019). Presepsin versus other biomarkers to predict sepsis and septic shock in patients with infection defined by Sepsis-3 criteria: the PREDI study of diagnostic accuracy. Emergencias.

[20] Lv D, Zhang K, Zhu C, Xu X, Gong H, Liu L (2024). Predictive value of CD86 for the occurrence of sepsis (Sepsis-3) in patients with infection. PLoS One.

[21] Lam RPK, Dai Z, Lau EHY (2024). Comparing 11 early warning scores and three shock indices in early sepsis prediction in the emergency department. World J Emerg Med.

[22] Arina P, Hofmaenner DA, Singer M (2024). Definition and Epidemiology of Sepsis. Semin Respir Crit Care Med.

[23] Stenberg H, Li X, Pello-Esso W (2023). The effects of sociodemographic factors and comorbidities on sepsis: A nationwide Swedish cohort study. Prev Med Rep.

[24] De Gaudio AR, Rinaldi S, Chelazzi C, Borracci T (2009). Pathophysiology of sepsis in the elderly: clinical impact and therapeutic considerations. Curr Drug Targets.

[25] Sun H, Hong Q, Li C (2025). Immunosenescence in Sepsis: Molecular Mechanisms and Potential Therapeutic Targets. Aging Dis.

[26] Ginde AA, Blatchford PJ, Trzeciak S (2014). Age-related differences in biomarkers of acute inflammation during hospitalization for sepsis. Shock.

[27] Rose N, Agrama I, Nachtigall I (2025). Sex differences in sepsis outcomes across the lifespan: a population-based cohort study in Germany. Crit Care.

